# Supplementary material for: The physiological basis for contrast opponency in motion computation in Drosophila
Source: Nat Commun. 2021 Aug 17;12:4987. doi: 10.1038/s41467-021-24986-w (PMC8371135; doi:10.1038/s41467-021-24986-w)
Supplement: Supplementary file 1 — Supplementary Information [file 41467_2021_24986_MOESM1_ESM.pdf]

The physiological basis for contrast opponency in motion detection in *Drosophila*

Supplementary information

Supplementary figures

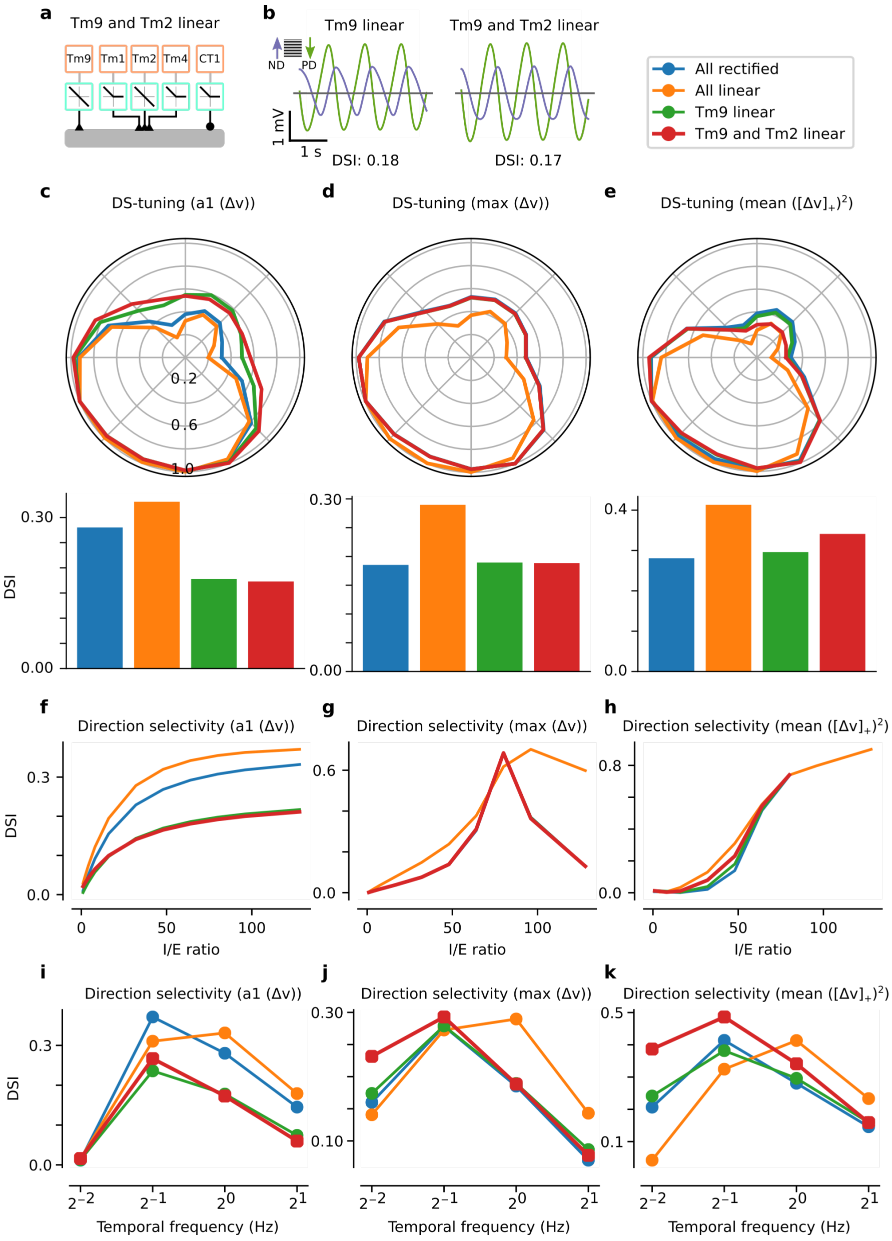

Supplementary Fig. 1: Comparison of models of local motion detection in *Drosophila* across read-outs, parameters, and temporal frequencies. Continued on next page.

Supplementary Fig. 1: Linear inputs benefit models of direction selectivity over a region of the parameter and stimulus space.. **a** Schematic showing a T5 model with a linear OFF input on the Tm9, and the Tm2 sites. **b** Voltage responses in the preferred and null directions (ND) to a sinewave grating from the Tm9 linear model (**Fig. 1g**), and the Tm9 and Tm2 linear model in **a**. The direction-selectivity index (DSI) is shown below the traces, calculated from the amplitude of the Fourier component at 1 Hz (a1 amplitude) of the voltage responses for each of the 16 directions of motion simulated. Model responses are to a grating with a spatial frequency of 24 deg per cycle and temporal frequency of 1 Hz (**b**-**h**). **c**-**e** Tuning curves across motion directions for models in **Fig. 1f** and in **a**. Top: Polar plots of response vs. direction of motion for the a1 amplitude of the voltage response (**c**), the maximum voltage response (**d**), and the mean calcium response (**e**), obtained by squaring the positively-rectified voltage response $([\Delta v]_+)^2$. Bottom: Direction-selectivity indices obtained from the tuning curves on the corresponding panels on top. **f**-**h** Direction-selectivity index vs. inhibition to excitation ratio (I/E) quantified using the a1 amplitude of the voltage response (**f**), the maximum voltage response (**g**), and the mean calcium response (**h**). For large I/E (above 80) responses are mostly negative, resulting in no calcium response. The all linear model consistently achieves higher DSI than the rest. **i**-**k** Direction-selectivity index vs. temporal frequency quantified using the a1 amplitude of the voltage response (**i**), the maximum voltage response (**j**), and the mean calcium response (**k**). Model responses are to a grating with a spatial frequency of 24 deg per cycle, moving downwards with temporal frequencies of 0.25 Hz, 0.5 Hz, 1 Hz, and 2 Hz. The all linear or the Tm9 and Tm2 linear models almost consistently achieves higher DSI than the rest.

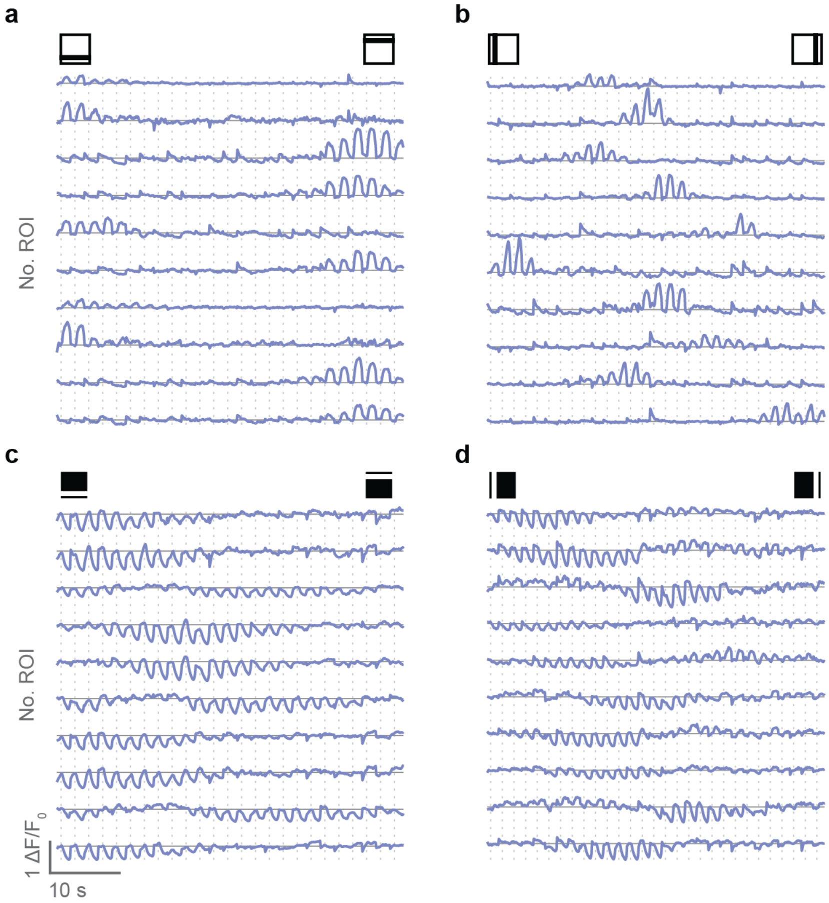

Supplementary Fig. 2: Tm9 calcium traces. Calcium traces of ten randomly selected Tm9 example neurons to horizontal and vertical OFF (**a, b**) or ON (**c, d**) bars. Neurons from the dataset shown in **Fig. 2** were randomly selected for each stimulus. For each bar position, the calcium response ranging from 0.5 s before the appearance of the bar (1 s) to 0.5 s after the disappearance of the bar was trial averaged. Calcium traces are concatenated matching the spatial order of the bar. In the stimulus presentation, the order of the bar presentations was pseudo-randomized. Source data are provided as a Source Data file.

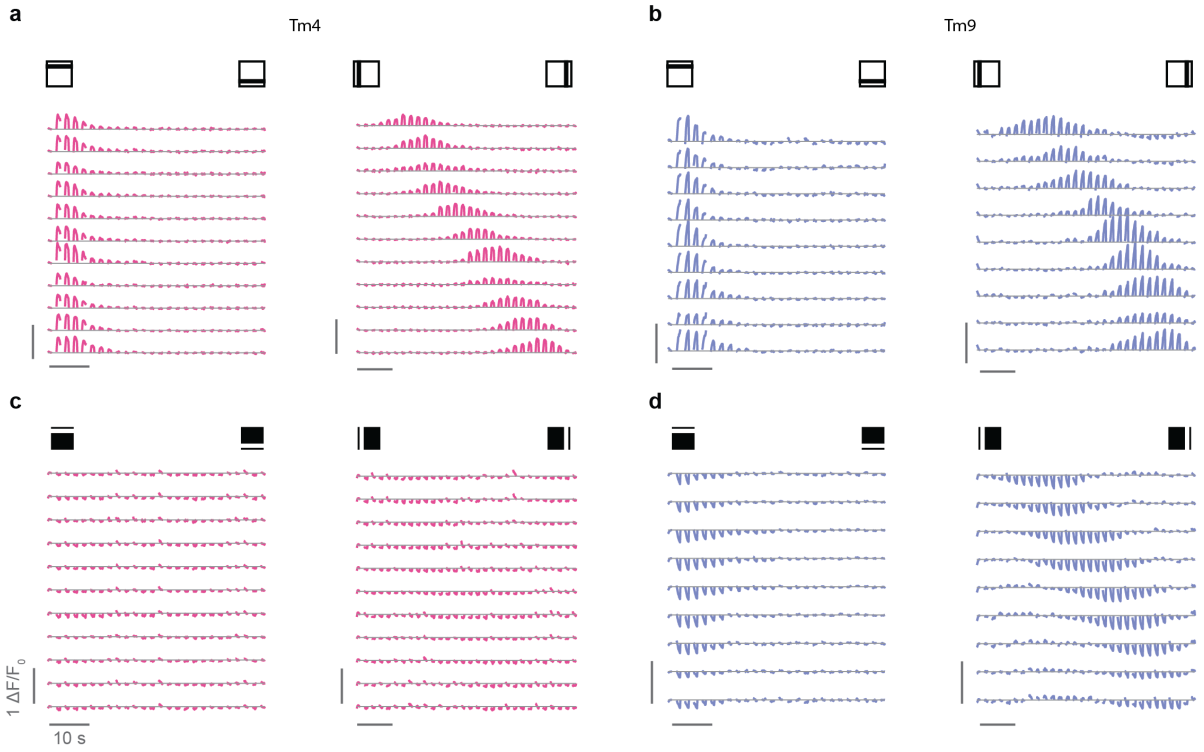

Supplementary Fig. 3: Tm9 and Tm4 calcium traces from the same fly. Calcium traces of Tm4 (**a, c**) and Tm9 (**b, d**) example neurons imaged simultaneously in one fly, responding to horizontal and vertical OFF (**a, b**) or ON (**c, d**) bars. For each bar position, the calcium response during the 1 s presentation of the bar was trial averaged. Calcium traces are concatenated matching the spatial order of the bar. In the stimulus presentation, the order of the bar presentations was pseudo-randomized. Source data are provided as a Source Data file.

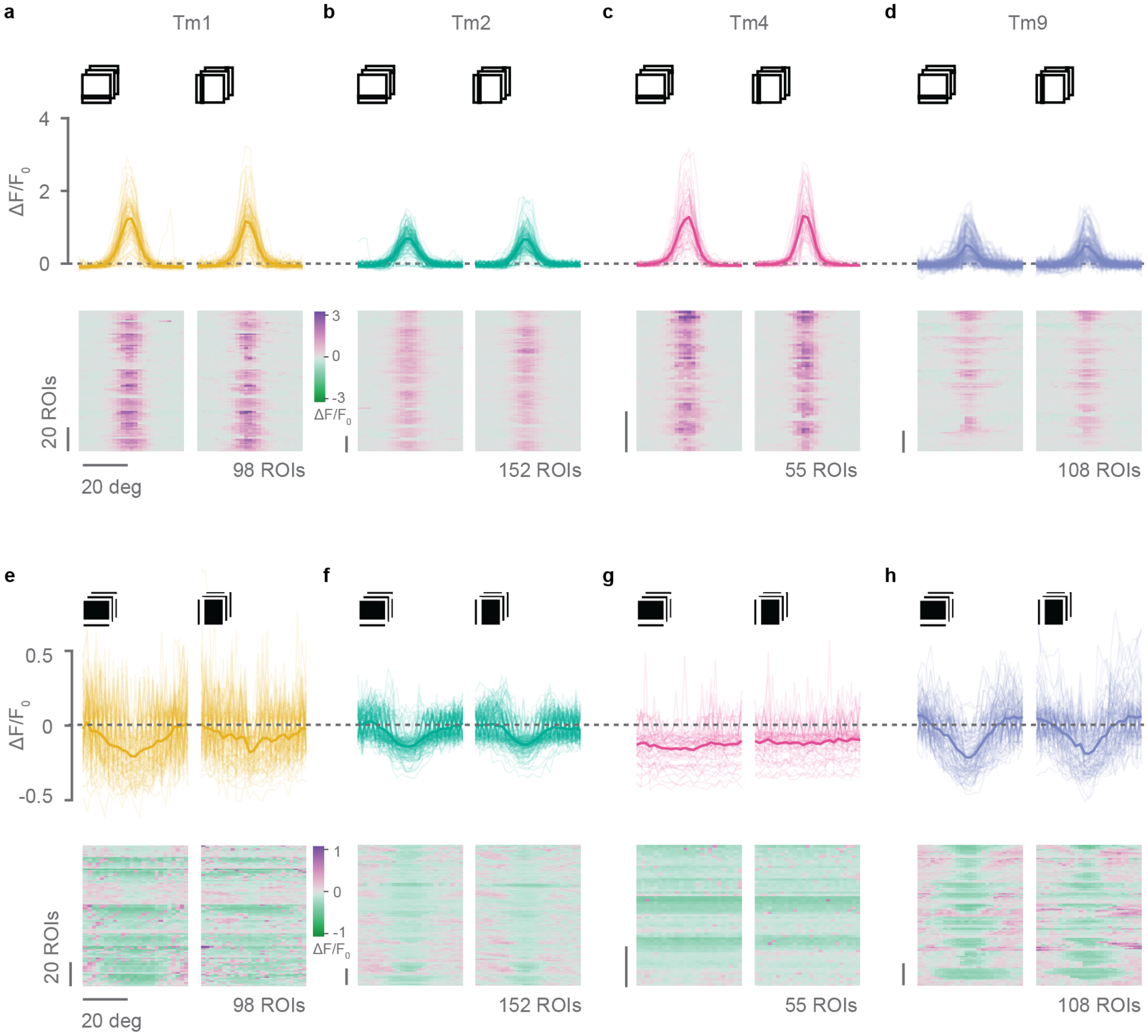

Supplementary Fig. 4: Receptive fields of Tm1, Tm2, Tm4 and Tm9 neurons. **a-d** OFF receptive fields obtained from *in vivo* two-photon calcium imaging of responses to horizontal and vertical dark bars from Tm1 (**a**), Tm2 (**b**), Tm4 (**c**), and Tm9 (**d**) neurons expressing GCaMP6f. This shows all recorded neurons, from which a subset with responses in both orientations fitted by a single Gaussian with $r^2 > 0.2$ and response quality index above 0.5 were included in **Fig. 4**. Number of ROIs (neurons, thin lines) is shown below the plots on the right. Mean across ROIs are thick lines. **e-h** ON receptive fields obtained from responses to horizontal and vertical bright bars from Tm1 (**e**), Tm2 (**f**), Tm4 (**g**), or Tm9 (**h**) neurons in **a-d**. Only Tm2 and Tm9 neurons had ON receptive fields visible averaging all recorded neurons without any selection criteria. This shows all recorded neurons, from which a subset with responses in both orientations fitted by a single Gaussian with $r^2 > 0.2$ were included in **Fig. 4**. Source data are provided as a Source Data file.

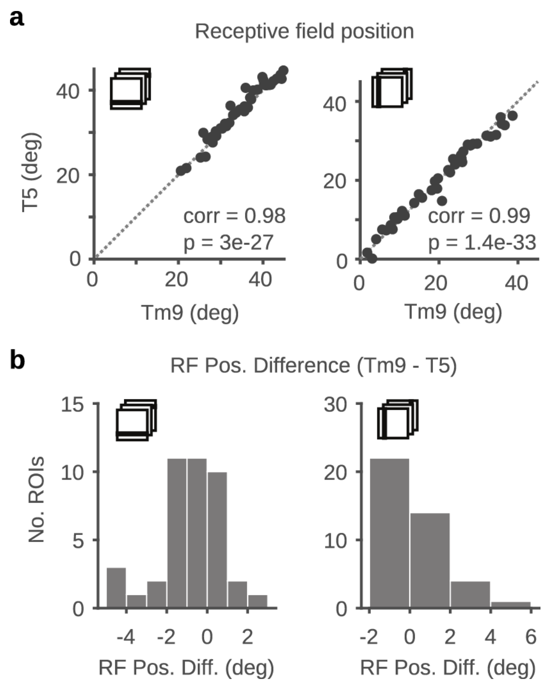

Supplementary Fig. 5: Receptive field positions are closely matched for overlapping T5 and Tm9 ROIs. **a** Scatter plot of receptive field positions obtained from Gaussian fits to Tm9 and T5 tuning curves in response to OFF bars shown in **Fig. 6b, d**. Pearson's correlations (corr), and associated two-tailed p-value (p), n = 41 ROIs (regions of interest). **b** Distribution of receptive field position differences between overlapping Tm9 and T5 ROIs in **a**, showing receptive fields overlap in visual space. Source data are provided as a Source Data file.

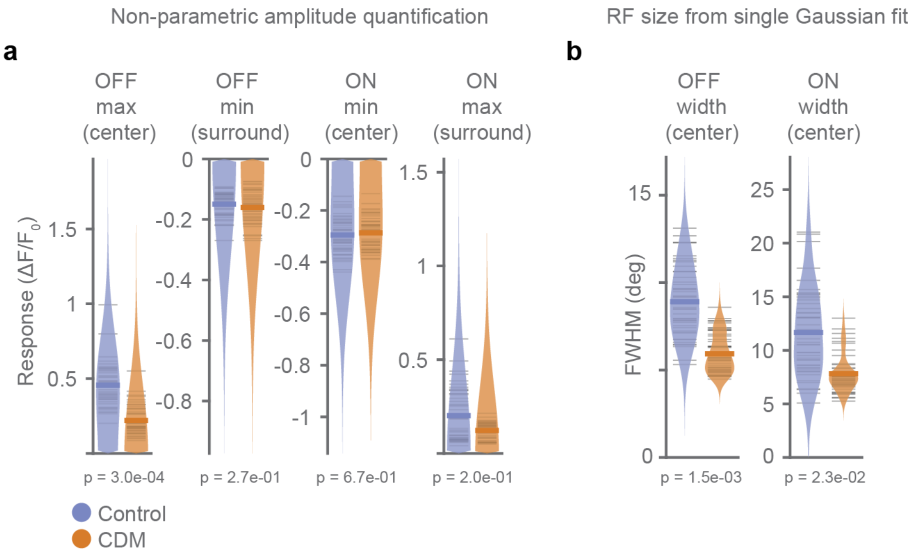

Supplementary Fig. 6: Chlordimeform (CDM) effects on Tm9 receptive fields. **a** Non-parametric quantification of the approximate receptive field center and surround amplitudes, respectively defined by the maximum and minimum values for positive OFF receptive fields, and as the minimum and maximum responses for the negative ON receptive fields. Data points correspond to the ones used in **Fig. 7a-e**. **b** Receptive field full width at half maximum (FWHM) obtained from a single Gaussian fit (to data in **Fig. 7a, b**) to quantify sizes of the net receptive fields. Each point is the average of the minimum/maximum amplitude (**a**) or fit FWHM (**b**) of a neuron across orientations. Two-tailed permutation tests comparing controls and CDM, p-values are indicated. Source data are provided as a Source Data file.
