# Supplementary material for: The physiological basis for contrast opponency in motion computation in Drosophila
Source: Nat Commun. 2021 Aug 17;12:4987. doi: 10.1038/s41467-021-24986-w (PMC8371135; doi:10.1038/s41467-021-24986-w)
Supplement: Supplementary file 3 — Reporting Summary [file 41467_2021_24986_MOESM3_ESM.pdf]

nature research

Corresponding author(s): Marion Silies

Last updated by author(s): Jul 5, 2021

Reporting Summary

Nature Research wishes to improve the reproducibility of the work that we publish. This form provides structure for consistency and transparency in reporting. For further information on Nature Research policies, see our Editorial Policies and the Editorial Policy Checklist.

Statistics

For all statistical analyses, confirm that the following items are present in the figure legend, table legend, main text, or Methods section.

n/a	Confirmed	
☐	☒	The exact sample size (n) for each experimental group/condition, given as a discrete number and unit of measurement
☐	☒	A statement on whether measurements were taken from distinct samples or whether the same sample was measured repeatedly
☐	☒	The statistical test(s) used AND whether they are one- or two-sided<br>*Only common tests should be described solely by name; describe more complex techniques in the Methods section.*
☐	☒	A description of all covariates tested
☒	☐	A description of any assumptions or corrections, such as tests of normality and adjustment for multiple comparisons
☐	☒	A full description of the statistical parameters including central tendency (e.g. means) or other basic estimates (e.g. regression coefficient) AND variation (e.g. standard deviation) or associated estimates of uncertainty (e.g. confidence intervals)
☐	☒	For null hypothesis testing, the test statistic (e.g. F, t, r) with confidence intervals, effect sizes, degrees of freedom and P value noted<br>*Give P values as exact values whenever suitable.*
☒	☐	For Bayesian analysis, information on the choice of priors and Markov chain Monte Carlo settings
☒	☐	For hierarchical and complex designs, identification of the appropriate level for tests and full reporting of outcomes
☐	☒	Estimates of effect sizes (e.g. Cohen's d, Pearson's r), indicating how they were calculated

Our web collection on statistics for biologists contains articles on many of the points above.

Software and code

Policy information about availability of computer code

Data collection	Prairie View (5.4) for imaging data acquisition.
Data analysis	Matlab 2017b for data analysis and plotting (https://gin.g-node.org/GRamosT/off-motion-receptive-fields), Python 3.5 and NEURON 7.4 for modeling (https://github.com/silieslab/RamosTraslosheros_Silies_2021)

For manuscripts utilizing custom algorithms or software that are central to the research but not yet described in published literature, software must be made available to editors and reviewers. We strongly encourage code deposition in a community repository (e.g. GitHub). See the Nature Research guidelines for submitting code & software for further information.

Data

Policy information about availability of data

All manuscripts must include a data availability statement. This statement should provide the following information, where applicable:

- Accession codes, unique identifiers, or web links for publicly available datasets
- A list of figures that have associated raw data
- A description of any restrictions on data availability

Figures 2-7 and Supplementary Figures 2-6 contain experimental data provided as a Source Data excel file. The processed MATLAB files are in a public g-node repository with DOI:10.12751/g-node.qeeyfz together with the code to plot figures and export the Source Data (https://gin.g-node.org/GRamosT/off-motion-receptive-fields)

Field-specific reporting

Please select the one below that is the best fit for your research. If you are not sure, read the appropriate sections before making your selection.

- [x] Life sciences
- [ ] Behavioural & social sciences
- [ ] Ecological, evolutionary & environmental sciences

For a reference copy of the document with all sections, see nature.com/documents/nr-reporting-summary-flat.pdf

Life sciences study design

All studies must disclose on these points even when the disclosure is negative.

Sample size	Sample sizes were chosen without calculations based on previous literature sample sizes, recording at least 5 flies and 30 neurons per condition to allow for statistical analysis. Individual neurons (regions of interest) are the unit of sample size. Previous studies used flies in the range from 3 to 17 (Serbe et. al (2016) Neuron, Strother et. al (2017) Neuron, Fisher et al. (2015) Curr. Bio., Badwan et. al (2019) Nat. Neuro.). On average our datasets contain about 10 flies, and lie within the range reported in the literature.
Data exclusions	No data was excluded for analysis, except when recordings were aborted due to strong motion of the living fly. Receptive field analysis fitted Gaussians to tuning curves, and non-responsive neurons were not well fit and did not appear in the final figures, although they form part of the dataset, and some are shown in the supplements.
Replication	All recordings contain recordings of flies from different food vials. Crosses were set up with 1-3 males and 5-10 females. Internal replication of data regarding Tm9 ON receptive fields is offered by independent datasets in figs. 2-6. Tm4 properties are replicated in figs. 3,4. All attempts at replication were succesful.
Randomization	Experimental groups were determined by the genotype of the flies, flies of different genotypes were not randomized but recorded during similar time frames using the same experimental protocol. Stimulus presentation trials were randomized.
Blinding	The investigators were not blinded to the fly genotypes, the same experimental procedure applied to all groups within a dataset.

Reporting for specific materials, systems and methods

We require information from authors about some types of materials, experimental systems and methods used in many studies. Here, indicate whether each material, system or method listed is relevant to your study. If you are not sure if a list item applies to your research, read the appropriate section before selecting a response.

Materials & experimental systems

n/a	Involved in the study	
x		Antibodies
x		Eukaryotic cell lines
x		Palaeontology and archaeology
	x	Animals and other organisms
x		Human research participants
x		Clinical data
x		Dual use research of concern

Methods

n/a	Involved in the study	
x		ChIP-seq
x		Flow cytometry
x		MRI-based neuroimaging

Animals and other organisms

Policy information about studies involving animals; ARRIVE guidelines recommended for reporting animal research

Laboratory animals	Drosophila melanogaster females from 1 to 7 days post eclosion were used in this study.
Wild animals	The study did not involve wild animals.
Field-collected samples	The study did not involve field-collected samples.
Ethics oversight	No ethical approval was required for fly experiments.

Note that full information on the approval of the study protocol must also be provided in the manuscript.
